# Emerging Methods for Enhancing Pluripotent Stem Cell Expansion

**DOI:** 10.3389/fcell.2020.00070

**Published:** 2020-02-14

**Authors:** Sarah W. Chan, Muhammad Rizwan, Evelyn K. F. Yim

**Affiliations:** ^1^Department of Chemical Engineering, Faculty of Engineering, University of Waterloo, Waterloo, ON, Canada; ^2^Waterloo Institute for Nanotechnology, University of Waterloo, Waterloo, ON, Canada; ^3^Centre for Biotechnology and Bioengineering, University of Waterloo, Waterloo, ON, Canada

**Keywords:** pluripotent stem cell culture, mechanobiology, three-dimension (3D) culture methods, topography, stiffness, encapsulation, microcarriers, suspension

## Abstract

Pluripotent stem cells (PSCs) have great potential to revolutionize the fields of tissue engineering and regenerative medicine as well as stem cell therapeutics. However, the end goal of using PSCs for therapeutic use remains distant due to limitations in current PSC production. Conventional methods for PSC expansion have limited potential to be scaled up to produce the number of cells required for the end-goal of therapeutic use due to xenogenic components, high cost or low efficiency. In this mini review, we explore novel methods and emerging technologies of improving PSC expansion: the use of the two-dimensional mechanobiological strategies of topography and stiffness and the use of three-dimensional (3D) expansion methods including encapsulation, microcarrier-based culture, and suspension culture. Additionally, we discuss the limitations of conventional PSC expansion methods as well as the challenges in implementing non-conventional methods.

## Introduction

**FIGURE 1:**
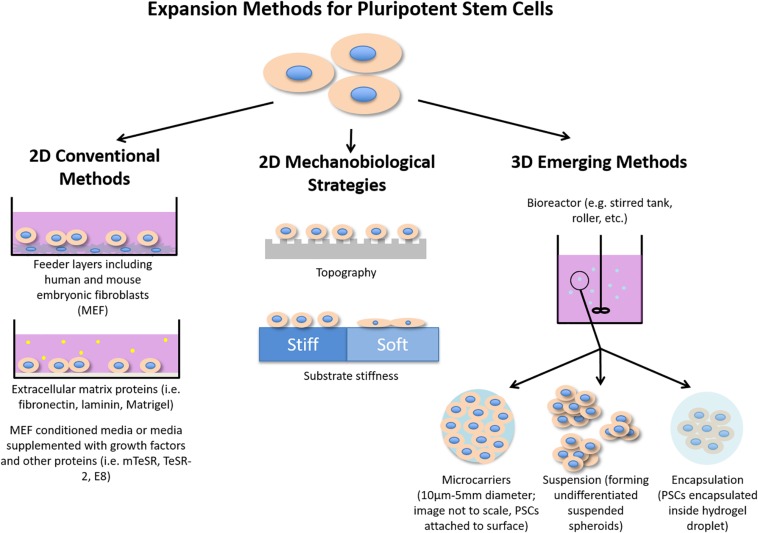
Illustration summarizing conventional methods of PSC expansion as well as mechanobiological strategies and emerging 3D methods for enhancing PSC expansion.

Pluripotent stem cells (PSCs), including embryonic and induced pluripotent stem cells (ESCs and iPSCs, respectively), are unique for their unlimited self-renewal and ability to differentiate into any cell of the three germ layers. These potentials could revolutionize the fields of disease modeling and regenerative medicine. Conventional PSC expansion methods, including feeder layers and the addition of growth factors to feeder-free culture, have been shown to maintain the undifferentiated state of PSCs efficiently. However, using feeder layers to expand human PSCs (hPSCs) is limited by concerns of transmission of animal pathogens and immunogens for clinical applications (Villa-Diaz et al., 2013) and are laborious to work with, having to culture two types of cells. Additionally, both methods can be irreproducible due to the poorly defined xenogenic culture conditions. Although xeno-free and defined media for hPSC expansion (Chen G. et al., 2011; Baghbaderani et al., 2016; Yasuda et al., 2018) are available, such media are expensive to scale-up for clinical use (Chen et al., 2014). Thus, much research has gone into novel methods that can improve hPSC expansion such as using mechanobiological principles, including surface topography, stiffness and surface modification. Mechanobiological principles have shown promises in reducing or replacing the need for biochemical growth factors in PSC culture (Ireland and Simmons, 2015; Argentati et al., 2019). For example, the transforming growth factor-beta (TGF-β) pathway, which is essential to maintaining hPSC pluripotency (James et al., 2005), can be activated by mechanotransduction, eliminating the need for supplementing TGF-β (Eyckmans et al., 2011; Rys et al., 2016). Use of the synthetic PSC niche is motivated by their low cost and high availability (Brafman et al., 2010; Fan et al., 2015). This review will focus on two types of emerging methods for improving PSC expansion: (1) two-dimensional (2D) methods that employ mechanobiological principles (e.g. topography and stiffness) and (2) three-dimensional (3D) methods of expansion including use of encapsulation, microcarriers, and suspension. Figure 1 summarizes both conventional and emerging strategies for enhancing PSC expansion.

As the field is not yet mature, the majority of studies have used mouse models as groundwork for human PSC studies. It is noteworthy, however, that results are not necessarily consistent between the two species due to differences in pathways associated with maintenance and the state of pluripotency of the cell. Mouse ESCs (mESCs) are in the naïve state of pluripotency, in which there has been no lineage specification (Ying et al., 2008); while hPSCs are in the primed state of pluripotency after isolation from the blastocyte (Huang et al., 2012), though generation of naïve hPSCs has been recently achieved (Zimmerlin et al., 2016; Yang et al., 2017; Lipsitz et al., 2018) with much of the knowledge gained from studying mESCs. Although the overall goal for improvement of PSC expansion, we will discuss mPSC studies in addition to hPSC studies to highlight the importance of mechanobiology in regulating PSC fate as most work in mechanobiology relating to PSC expansion has been done in mouse PSCs (mPSCs). Due to the differences in pluripotency states, the numerous differences in patterns of pluripotency-associated gene expression, morphology, culture requirements, differentiation behavior and molecular profiles will determine different expansion methods for mPSCs and hPSCs (Nichols and Smith, 2009; Davidson et al., 2015). We suggest an excellent review by Davidson et al. (2015) for a comprehensive understanding of the differences and significances of mouse and human pluripotency.

## Conventional Methods of PSC Expansion

PSCs are commonly cultured using feeder layers or feeder-free systems (Table 1) that require the use of a biological matrix supplemented with chemical growth factors. Feeder layers consist of cells that create and maintain the stem cell niche required for expanding and maintaining the pluripotency of PSCs (Johnson et al., 2008). Feeder cells provide the biochemical factors required by PSCs for self-renewal and proliferation, along with biophysical cues, including topography and stiffness (López-Fagundo et al., 2016). We suggest a comprehensive review on feeder layers by Llames et al. (2015).

**TABLE 1:** A summary of conventional methods of PSC expansion with their advantages and limitations.

Method	Description	Advantages	Limitations
Mouse embryonic fibroblast (MEF) Feeder Layer	Uses mitotically inactivated MEF cells, treated with gamma irradiation or mitomycin C (Conner, 2000), such as SNL and STO lines The most traditional method of maintaining and expanding PSC; used by Kaufman and Evans (1981) for the first mouse embryonic stem cell (mESC) culture, and then by Thomson et al. (1998) for the first human embryonic stem cell (hESC) culture as well as in Takahashi and Yamanaka (2006) for the first induced pluripotent stem cell (iPSC) culture and in Takahashi et al. (2007) and Yu et al. (2007) for the first human iPSC (hiPSC) cultures	Commonly used Inexpensive Well-documented	Xenogenic Difficult to scale up expansion into 3D Laborious Two cultures Undefined components Batch to batch variation (Amit and Itskovitz-Eldor, 2006)
Human feeder layer	Uses human cells, such as fetal fibroblasts (Richards et al., 2002), adult fallopian tube epithelial cells (Richards et al., 2002), foreskin fibroblasts (Hovatta et al., 2003; Amit et al., 2004; Yang et al., 2016), and autologous dermal fibroblasts (Takahashi et al., 2009), to create and maintain the stem cell niche by providing the biochemical growth factors and biophysical cues required for expanding and maintaining the pluripotency of PSC	Commonly used Inexpensive Well-documented Xeno-free	Cannot up-scale expansion into 3D Laborious Two cultures Chemically undefined Batch to batch variation (Amit and Itskovitz-Eldor, 2006)
MEF-conditioned media	Produced by incubating hPSC media (DMEM/F12, Knockout Serum Replacement, L-glutamine, non-essential amino acids, and β-mercaptoethanol) with MEF overnight (Tomishima, 2014) or can be purchased commercially Fibroblast growth factor 2 (FGF2) is added to the media before use	Feeder free Well-documented	Chemically undefined Xenogenic components Batch to batch variation
Essential 8 media	Used with a vitronectin coating for a defined culture Only the essential 8 factors for hPSC propagation: DMEM/F12, human insulin, human transferrin, selenium, ascorbic acid, sodium hydrogen carbonate, human recombinant FGF2, and transforming growth factor b (or NODAL) (Chen G. et al., 2011)	Xeno-free Chemically defined Feeder free Less expensive	Inconsistent and less robust than mTeSR (Hey et al., 2018) More laborious Slow growth rates Higher radical oxygen species (ROS) resulting in increased genotoxic stress (Bangalore et al., 2017)
mTeSR media	Currently the most common system used for hPSC expansion Typically used with a Matrigel ECM coating Contains bovine serum albumin	Feeder free Well-documented Easy to use Less laborious Robust and consistent	Xenogenic components Not completely defined Matrigel is undefined and may vary from batch to batch Higher radical oxygen species (ROS) resulting in increased genotoxic stress (Bangalore et al., 2017)
TeSR-2 media	Used with vitronectin or laminin 521 coatings for completely defined, xeno-free culture system	Xeno-free Feeder free Chemically defined Well-documented Easy to use Less laborious	Expensive and costly (Chen G. et al., 2011)

The other conventional methods of culturing PSCs involve using ECM components with cell culture media supplemented with growth factors that regulate genes related to pluripotency (Srinivasan et al., 2016) – either to up-regulate promoters of pluripotency or down-regulate inhibitors of pluripotency. The growth factors used depend on the pathways to be regulated, which depend on the cell type. For example, mPSC culture depends on growth factors such as leukemia inhibitory factor (LIF) (Smith et al., 1988; Williams et al., 1988) to maintain pluripotency, while hPSC maintenance depends on fibroblast growth factor 2 (FGF2) (Dvorak et al., 2006) and Activin A (Beattie et al., 2005). Despite containing animal-derived products (bovine serum albumin), the most commonly used feeder-free media for hPSC expansion is mTeSR media and is typically used with the animal-derived Matrigel coating.

Xeno-free and chemically defined systems have been developed for PSC expansion. However, their high cost limits its use in large-scale production of PSCs (Chen G. et al., 2011). The most basic xeno-free medium for hPSC expansion is Essential 8 (E8). These media are used with a vitronectin coated culture vessel to make the expansion system completely defined and xeno-free. However, E8’s use in hPSC expansion is limited due to inconsistencies and slower growth rates (Hey et al., 2018). Therefore, it is worth exploring the use of physical and mechanical cues in PSC maintenance and expansion, which could improve the large-scale xeno-free expansion. We recommend a book chapter (Srinivasan et al., 2016) for a comprehensive review of conventional hPSC expansion, a review by Dakhore et al. (2018) that compares hPSC expansion media, and a review by Hayashi and Furue (2016) that summarizes substrates used in hPSC expansion.

## Non-Conventional Methods of PSC Expansion

Due to the limitations of conventional methods PSC expansion, new expansion methods that improve PSC expansion are needed to make progress toward therapeutic use of PSCs. Additionally, these non-conventional methods aim to improve efficiency, reproducibility and cost. For clinical use, current Good Manufacturing Practice (GMP) is an important aspect to consider. However, most mechanobiological studies of PSC expansion have not covered this area yet. We recommend the recent review by Bedford et al. (2018) for a comprehensive review of cGMP for cell therapy and the review by De Sousa et al. (2016), which reviews cGMP in hPSC expansion specifically.

### Two-Dimensional (2D) Non-conventional Methods

2D methods focus on surface and materials properties of the expansion substrate, which can scale-out expansion in 2D and be implemented into a 3D culture for scaling up expansion. The 2D mechanobiological strategies that have been studied include growth factor immobilization (Alberti et al., 2008; Sohi et al., 2018) and micropatterning with proteins (Mosiewicz et al., 2013; Hammad et al., 2016) or other ECM molecules (Meade et al., 2013), surface chemistry (Saha et al., 2011; Kimura et al., 2018), and nanomaterials including graphene (Chen G.Y. et al., 2012) and carbon nanotubes (Akasaka et al., 2011; Pryzhkova et al., 2014). However, we will only discuss topographical cues and stiffness in this review.

#### Topographical Cues for PSC Expansion

Topography plays a key role in determining PSC fate (Ankam et al., 2013, 2015, 2018; Chan et al., 2013), including maintaining pluripotency and regulating self-renewal and proliferation. Table 2 lists examples of studies of micro and nanotopographies on PSC maintenance and expansion. Studies of hPSCs suggest that smaller topographical features promote their undifferentiated state (Bae et al., 2014; Reimer et al., 2016; Ko et al., 2017). Using a TopoChip with over 1000 patterns made of tissue culture polystyrene (T), small feature size with high feature density were found to promote hPSC pluripotency best (Reimer et al., 2016). Comparatively, another study found that nano-pillars and nano-grooves of around 200 nm on polydimethylsiloxane (PDMS) promoted proliferation and maintenance of hiPSCs in feeder-free conditions (Ko et al., 2017). A study using nanopillars of 120–360 nm in diameter, found that pillars with diameters 120–170 nm retained the most pluripotency marker expression and had the least amount of colony spreading (Bae et al., 2014). Meanwhile, hESCs cultured on vitronectin-coated micro-patterns, binary colloidal crystals of 2 and 5 μm, resulted in improved maintenance of pluripotency (Wang et al., 2018), though this study did not involve nano-sized patterns. In contrast, Chen W. et al. (2012) compared hESC expansion on 150 nm rough glass surface to expansion on a smooth glass surface and found that Oct4 expression of hESCs was lower on the nanorough surface compared to the smooth surface.

**TABLE 2:** Summary of examples of studies of human or mouse pluripotent stem cells (hPSCs and mPSCs, respectively) on topographical features and their results.

Cell type	Feature type	Size	Substrate material and ECM coating	Media used	Characterizations performed	References
Mouse embryonic stem cell (mESC)	Hierarchically structured surfaces Hierarchically structured surfaces N/A	Micro-nano (MN) (9 μm height, 919 ± 22 nm average surface roughness) Nano (68 ± 30 nm average surface roughness) Smooth (2 ± 0.4 nm average surface roughness)	2-hydroxyethyl methacrylate-co-ethylene dimethacrylate (HEMA-EDMA) (no coating) HEMA-EDMA (no coating) HEMA-EDMA (no coating)	Leukemia inhibitory factor (LIF)-containing mESC media LIF-containing mESC media LIF-containing mESC media	Colony circularity increased in MN substrates compared to on feeder layers after 4 passages Western blot showed increased Oct4 and Nanog protein levels after 4 passages Higher percentage of OCT4 + compared to feeder layer control (Immunofluorescent (IF) imaging) Similar number of cells to feeder layer control Higher percentage of OCT4 + compared to feeder layer control (IF imaging) Fewer cells than on feeder layer control Higher percentage of OCT4 + compared to feeder layer control (IF imaging) Fewer cells than on feeder layer control	Jaggy et al., 2015
	Nanotopography	16 nm 38 nm 68 nm	Gold nanoparticles treated with allylamine, acrylic acid or octadiene and coated with fibronectin	mESC media	16 nm topography showed the lowest mean cell area; significantly less spreading and proliferation From IF imaging, all topographies maintained pluripotency gene expression (Oct4 and Nanog) after 72 h, except when treated with acrylic acid	Macgregor et al., 2017
	Roughness	Root-mean square average roughness less than 392 nm	Aminated gold nanoparticle layers	LIF-containing mESC media	Immunocytochemistry (ICC) for Oct4 showed positive in substrates with roughness less than 392 nm ICC for phalloidin and vinculin showed that nanoroughness supported focal adhesion formation while microroughness decreased focal adhesion formation MTT proliferation and viability assay showed higher proliferation rates in substrates with roughness less than 392 nm Alkaline phosphatase (ALP) activity significantly higher in substrates with roughness less than 392 nm than substrates with roughness greater than 573 nm Quantitative (q)-PCR showed no significant decrease in Oct4 expression in substrates with roughness less than 392 nm Reverse transcriptase (RT)-PCR for endoderm, mesoderm and ectoderm markers found in substrates with roughness more than 573 nm	Lyu et al., 2014
	Grooves Hexagonal Square pillar	Ridge 5 μm Ditch 15 μm Depth 5μm Ridge 5 μm side length 15 μm Depth 5μm Side length 10 μm Inter-pillar gap size 10 μm Depth 5 μm	Polyacrylamide hydrogel functionalized with collagen I	Mouse embryonic fibroblast (MEF)-conditioned media	IF images showed increased pluripotency (Nanog and Oct4) on hexagonal substrates compared to the smooth substrates Similar colony area on all substrates	Lü et al., 2014
	Nanofibres	Fiber diameter 550–750 nm	Polyethersulfone (PES) and collagen-grafted PES (PES-COL)	LIF-containing mESC media	RT-PCR for Oct4 and Nanog significantly higher on PES and PES-COL nanofibers compared to gelatin coated plates MTT proliferation assay showed PES-COL fibers had the significantly highest proliferation though PES fibers still had significantly higher proliferation than on gelatin coated plates Immunocytochemistry (ICC) for SSEA-1 and Oct4 showed the highest expression and dome shaped morphology in cells cultured on PES-COL fibers though cells on PES fibers still had significantly higher expression than on gelatin coated plates ALP assay showed the highest percentage of ALP in cells cultured on PES-COL fibers after 7 passages though cells on PES fibers still had significantly higher expression than cells cultured on gelatin coated plates, which spontaneously differentiated after 1–2 passages	Hashemi et al., 2011
	Nanofibers	N/A	Polyamide (Ultra-Web) coated with gelatin	LIF-containing mESC media	Colony sizes and proliferation rates of undifferentiated mESC were significantly larger on Ultra-Web substrates than on glass slides Rac activity was significantly higher in mESC cultured on Ultra-Web while Rho and Cdc42 activity was unchanged; implies that Rac is essential to mESC proliferation on nanofibrous substrates mESC cultured on Ultra-Web in the presence of retinoic acid expressed GFAP and Nestin while mESC cultured on Ultra-Web without retinoic did not express GFAP and Nestin; therefore, mESC cultured on Ultra-Web retain their ability to differentiate	Nur-E-Kamal et al., 2005
	Irregular nanopatterns	7–8 nm	Polydimethyl-siloxane (PDMS) coated with poly-D-lysine (PDL)	LIF-containing mESC media	Nanopatterned PDMS coated with PDL showed cell attachment and proliferation similar to on tissue culture polystyrene (TCPS) while flat PDMS showed low cell attachment ICC and q-RT-PCR showed increased expression of Oct4, Sox2, Nanog and Klf4 in cells cultured on nanopatterned PDMS Flow cytometry showed in cells cultured on nanopatterned PDMS expressed both Oct4 and SSEA-1 Phosphorylation of FAK, Src, JNK, c-Fos, and ERK decreased in cells cultured on nanopatterned PDMS, indicating that LIF and FAK pathways modulate upregulation of self-renewal-associated proteins and the suppression of spontaneous differentiation mESC were differentiated into the three germ cell lineages; the cells cultured on nanopatterned PDMS had a higher percentage of cells differentiated, thus these cells maintained a higher quality undifferentiated state	Jeon et al., 2012
	BioSurface structure assay; 504 different microstructures of square and round pillars	Alternating square and round pillars 1 μm laterally and 2 μm gap spacing	Silicon coated with 100 nm tantalum oxide layer	mESC media with and without LIF	Circular, well-defined compact colonies Cells passaged on this pattern produced 1 chimera with 100% germ line offspring and 4 sterile males, which was similar to the cells passaged on feeder layers Colonies were positive for Oct3/4 and Nanog	Markert et al., 2009
	Spheres	400 nm	Silica coated with collagen I	LIF-containing mESC media	Semi-quantitative PCR showed up-regulation of pluripotency markers and down-regulation of endoderm markers compared to on glass and in embryoid bodies Light interferometry showed reduced cell spreading on the silica spheres compared to on glass Scanning electron microscopy (SEM) formed rounder, more spherical colonies than on cover slips	Ji et al., 2012
Human embryonic stem cell (hESC)	Multi-architectural (MARC) chip; consists of gratings, pillars and holes	2 μm grating, 2 μm height, 2 μm spacing 2 μm grating, 120 nm height, 1 μm spacing 1 μm grating, 80 nm height, 2 μm spacing 250 nm grating, 250 nm height, 250 nm spacing 1 μm pillar, 6.5 μm pitch, 1 μm height 2 μm holes, 12 μm pitch, 2 μm height	PDMS coated with Matrigel PDMS coated with laminin	mTeSR1 medium Unconditioned hESC medium without supplements	ICC for Nanog showed maintained high levels of the pluripotency marker Nanog but low levels of Nestin ICC for Oct4 showed decreased Oct4 expression in 2 μm and 1 μm gratings, while the 250 nm gratings, 1 μm pillars and 2 μm holes had higher levels of Oct4	Ankam et al., 2013
	Binary colloidal crystals	2 μm silica particles and 0.11 μm PMMA 5 μm silica particles and 0.4 μm PMMA	Silica and polymethyl methacrylate (PMMA) with vitronectin coating	Essential 8 (E8) media	ICC showed that both surfaces of interest were positive for pluripotency markers Tra-1-60 and Oct4 Cells culture on the substrates of interest without a vitronectin coating were not maintained	Wang et al., 2018
	Nanopillars	120–360 nm with 400 nm spacing	Polystyrene (PS) coated with gelatin	hESC medium supplemented with 10 mM rho kinase (ROCK) inhibitor (removed after 2 days)	SEM images showed circular colony morphology on all pattern sizes, however, on the area with patterns 120–170 nm in size, there were less focal adhesions formed and less spreading ICC showed that patterns from 120 to 170 nm had the highest population of Oct4 + and SSEA4 + cells (93%) compared to on 170–190 nm patterns (79%) and 290–360 nm patterns (82%), though all were higher than on the flat control (53%) qPCR showed increased pluripotency marker (Oct4, Sox2, Nanog) expression in 120–290 nm sized pillar areas compared to the flat control; however, the 120–70 nm pillars had the significantly highest expression	Bae et al., 2014
	Microfiber	1.3 μm ± 0.25 μm fiber diameter	Polyurethane plasma treated with argon, hydrogen or oxygen	DEF-CS culture system (Cellectis)	ICC showed a large percentage of cells was Oct4 positive, showing retained pluripotency On randomly oriented fibers, all plasma treatments dramatically improved the expansion capability, as compared to the native fibers; increase in expansion was 7-fold for Ar fibers, 5-fold for H_2_ fibers and 4-fold for O_2_ fibers	Zandén et al., 2014
Human induced pluripotent stem cell (hiPSC)	Nanoroughness Smooth	Root-mean square average roughness 150 nm Root-mean square average roughness 1 nm	Glass coated with vitronectin	Human-Cell-Conditioned Medium (hCCM) with fibroblast growth factor 2 (FGF2)	hESCs adhered better to the smooth surface; a highly branched, filopodia-rich morphology of single hESCs was observed on the smooth surface compared to the more compact cells with few, short cytoplasmic extensions on the nano-rough surface using SEM	
					Oct3/4 expression was significantly better on the smooth surface (93%) compared to the rough surface (41%) On smooth glass focal adhesions formed on the periphery of Oct3/4 + cells with less spreading while on nanorough glass the focal adhesions formed randomly throughout the colony with more spreading	Chen W. et al., 2012
	Groove-ridge structures	200 nm height with 340, 650, and 1400 nm periodicity; height ∼70 nm	PDMS or polyimide (PI) coated with vitronectin	E8	Grooves with periodicity of 650and 1400 nm resulted in significant colony elongation compared to the control qPCR showed a significant decrease in Oct4 expression in cells cultured on the PI 650 nm grooves compared to the flat PI Upon bone morphogenetic protein (BMP) 4 stimulation, YAP reveals nuclear localization at the rim, whereas it is cytoplasmic at the center of differentiating iPSC colonies TAZ (a YAP paralog) strongly co-localizes with actin filaments and cell-material adhesion sites in iPSCs	Abagnale et al., 2017
	TopoChip (2176 patterns made of circles, rectangles and triangles)	Various, 10 μm height for all	TCPS	E8 with ROCK inhibitor	100 topographies were ranked top or bottom on the basis of number of Oct4 + cells at 24 h After 4 days, the top 100 topographies supported formation of extensive colonies of undifferentiated iPSC that expressed Oct4 and Sox2 At 24 h patterns with the greatest number of cells also had the greatest number of EdU + cells and Oct4 + cells, indicating that they supported self-renewal and prevented differentiation of iPSCs Computational analysis showed that small feature size and high feature density were most important in determining pluripotency	Reimer et al., 2016
	Irregular patterned nanofeatures Grooves Pillars	< 1 μm 100 nm width, 300 nm depth, 300 nm separation 300 nm diameter, 100 nm separation	PDMS coated with PLO and fibronectin	mTeSR1 medium supplemented with 10 mM ROCK inhibitor (removed after 2 days)	a-actinin expression was significantly greater in grooves and pillars than in flat and irregularly patterned surfaces at P6 and P10 Gene and protein expression of FAK did not change during passaging, except for an increase on grooves and pillars at P6. Ki67 (cell proliferation marker) significantly increased on grooves and pillars compared to on irregular nanofeatures and smooth surfaces ICC for Nanog and Oct3/4 significantly increased on grooves and pillars compared to PSCs on irregular nanofeatures and smooth surfaces SEM images showed that cells on grooves and pillars had fewer filopedia and more globular appearance than cells on irregular nanopatterns and smooth surfaces	Ko et al., 2017
hESC and hiPSC	Nanofiber	270 nm fiber diameter, density 4.6 μg/cm^2^	Gelatin	mTeSR1 medium	After 20 passages, cells grown on nanofibers continued expressed pluripotency markers Oct4, Nanog and Sox2 but not differentiation markers Pax6, Brachyury, and Afp; expression similar to cells cultured on Matrigel Flow cytometry showed high percentage of SSEA-4 + cells after being cultured on nanofibers qPCR of all genes (84) in the integrin family were analyzed; integrin expression did not change between culture conditions – both on nanofibers and on Matrigel – and had high levels of α5, α6, α7, αv, β1, and β5 and low expression of a8, which was high in cells cultured on flat gelatin *In vitro* embryoid body formation and *in vivo* teratoma formation was performed and cells cultured on the nanofibers were able to differentiate into cells of all three germ layers	Liu et al., 2014

While topography undoubtedly contributes significantly to PSC maintenance, a common consensus on which topographies are the most important for PSC maintenance has not been reached. Studies have implied that surface topography alone cannot maintain pluripotency (Ankam et al., 2013; Abagnale et al., 2017; Wang et al., 2018). Interestingly, in studies of mPSCs and hPSCs, it was hypothesized that topography affects focal adhesion formation, which affects colony morphology and stem cell fate. Colonies with retained pluripotency and compact, circular morphology on patterned areas showed significantly fewer focal adhesions compared to the colonies that spread out and grew in irregular shapes on flat surfaces (Jeon et al., 2012; Ji et al., 2012; Bae et al., 2014; Ko et al., 2017; Macgregor et al., 2017). Surface topography may prevent focal adhesion formation, which reduces spreading and leads to the compact, circular colonies associated with preserved pluripotency (Hashemi et al., 2011; Jeon et al., 2012; Ji et al., 2012; Kong et al., 2013; Bae et al., 2014; Ko et al., 2017). It has also been hypothesized that topography affects ECM protein adsorption, affecting the cell adhesion, proliferation and morphology, thus synergistically maintaining PSCs (Zandén et al., 2014; Macgregor et al., 2017).

#### Stiffness of Substrate on PSC Expansion

Substrate stiffness plays a significant role in controlling cellular behavior and stem cell fate. Synthetic biomaterials, including hydrogels, are useful tools for studying the effects of stiffness. Hydrogels can be modified to have different stiffness, depending on the molecular weight or concentration of polymer, and the crosslinking density (Caliari and Burdick, 2016), which depends on crosslinker concentration and crosslinking time. Polyacrylamide (PA) hydrogels (Pelham and Wang, 1997; Mih et al., 2011) and polydimethylsiloxane (PDMS) (Evans et al., 2009; Sun et al., 2012), with large tunable ranges of stiffness are examples used for studying the impact of stiffness on PSCs.

Mouse ESCs were cultured on PDMS substrates with varying stiffness and showed increased cell spreading and proliferation with increasing substrate stiffness (0.041–2.7 MPa) along with increased differentiation (Evans et al., 2009). Similarly, mESCs cultured on a stiff poly-L-lysine/hyaluronic acid (PLL/HA) hydrogel film showed increased cell adhesion and proliferation, while weak cell adhesion, and round colonies retaining pluripotency were observed on the softer PLL/HA films (Blin et al., 2010). Soft PA substrates maintained mESC pluripotency better than stiff substrates including TCPS (Chowdhury et al., 2010a). When cultured on substrates similar to mESC intrinsic stiffness (0.5–0.6 kPa), mESCs had improved self-renewal and retention of pluripotency, thus proposing stiffness matching as a method for maintaining mESCs (Chowdhury et al., 2010b). Later, mESCs cultured on PA hydrogels of varying stiffness preserved their pluripotency regardless of the surface topography, with increased Oct4 and Nanog expression on all soft substrates compared to the stiff substrates; topography only influenced pluripotency on cells cultured on stiff substrates (Lü et al., 2014).

The effects of stiffness observed in mESCs are not translated to hPSCs. There is limited consensus on the effects of stiffness, with some groups finding little to no influence of stiffness on pluripotency (Keung et al., 2012; Maldonado et al., 2015; Przybyla et al., 2016; Price et al., 2017) and others finding the opposite (Musah et al., 2012; Sun et al., 2012; Kim et al., 2018; Sung et al., 2018). Nonetheless, observations made by several groups imply that stiffer substrates are more suitable for hPSC expansion. Using PDMS with effective moduli of 1.92 kPa (soft), 14.22 kPa (medium rigid), and 1218.4 kPa (rigid), hESC cytoskeleton contractility was found to increase with matrix rigidity along with maintained pluripotency (Sun et al., 2012). Consistent with these findings, hESCs and hiPSCs, cultured on PA hydrogels functionalized with a glucosaminoglycan binding peptide, attached better and formed more spread out and robust colonies on substrates coated with and without a Matrigel coating (Musah et al., 2012). The stiffest substrates (10 kPa) were found to support hESC expansion in five different hESC lines, with high levels of YAP/TAZ nuclear localization, an indicator of pluripotency. Later studies also found that YAP/TAZ nuclear localization decreases in soft substrates (Price et al., 2017; Lee et al., 2019). Although YAP/TAZ nuclear localization decreased in soft substrates, pluripotency marker expression remained similar between soft and stiff substrates with higher proliferation in stiff substrates (Price et al., 2017). Similarly, as substrate stiffness increased, cell proliferation increased, and substrate stiffness has an inverse relationship with spontaneous differentiation (Maldonado et al., 2015). Ligand density also affects how cells respond to their substrate stiffness; with the right number of functional groups, soft materials can support hPSC attachment proliferation and self-renewal similar to that of a stiff hydrogel (Lee et al., 2019). Despite the inconclusiveness of exactly how substrate stiffness affects hPSC behavior, especially as material choice also affects pluripotency and the absolute stiffness required for PSC culture, it is clear that stiffer substrates are more suitable for hPSC expansion in contrast with softer substrates for mPSC expansion.

### Three-Dimensional (3D) Methods of PSC Expansion

Recently, 3D methods of stem cell culture have gained traction due to the need for scalable stem cell expansion to obtain therapeutically relevant number of cells. 3D cell culture methods offer the opportunity to significantly scale-up the expansion of hPSCs. The 3D methods of PSC culture are divided into three categories: (i) PSC encapsulation in hydrogels, (ii) microcarrier-based 3D PSC culture, and (iii) PSC suspension culture.

A growing body of literature suggests that 3D cell culture systems recapitulate the *in vivo* microenvironment of cells that could help to improve stem cells expansion. In a landmark study of PSC encapsulation, a defined and scalable 3D cell encapsulation system in a thermo-responsive hydrogel was developed for hPSC expansion and differentiation; it also enabled efficient retrieval of the cells from the hydrogels following expansion, without using the cell dissociation enzymes (Lei and Schaffer, 2013). The cells expanded ∼80-fold in 3D culture compared to ∼9-fold expansion in 2D over 15 days. Cumulatively, 3D cell culture led to 10^72^-fold expansion over 60 passages (Lei and Schaffer, 2013). PSCs are mechanosensitive as previously discussed; however, the role of biophysical signals and cell-matrix interactions in the context of 3D PSC expansion was not investigated. Scaffolds used for 3D expansion of PSCs should provide a balance between cell–cell contact and cell–matrix interactions (Li et al., 2012). An alginate-based hydrogel with tethered polypeptides comprising of a cell-binding sequence of E-cadherin for 3D PSC expansion has shown to improve the proliferation rate of PSCs without compromising pluripotency marker expression resulting in up to 23-fold higher expansion in HAV10 peptide conjugated gels (Banerjee et al., 2018). Matrix degradability and remodeling by encapsulated PSCs are other parameters that affect PSC fate. Encapsulated PSCs are known to remodel their environment during proliferation and differentiation (Khetan et al., 2013; Madl et al., 2017). However, the capacity of PSCs to remodel the environment and their effect on the pluripotency related markers are poorly understood. This should be studied in greater detail to design more informed and tailor-made 3D scaffolds for enhanced stem cell expansion.

Microcarrier-based systems are another method for PSC expansion, which combine 2D cell adhesion in microcarriers with a 3D configuration of the bioreactor system to expand the area available for PSC expansion. Microcarriers act as supporting substrates for adherent cell culture with a diameter varying from 10 μm up to 5 mm (Le and Hasegawa, 2019). The major benefit of using microcarriers is their capacity to provide large surface areas for cell growth while being compatible with adherent cell culture systems. Cells can form a confluent layer around the microporous microcarrier; while in macroporous microcarriers, they are entrapped inside the pores of the microcarriers (Badenes et al., 2014). Factors including the type of materials used to fabricate the microcarriers, the shape of the microcarrier, and the type of ECM coating used for cell adhesion influence the yield and pluripotency of the PSC culture in a microcarrier system (Chen A.K.L. et al., 2011). For instance, use of matrigel coating led to up to 18-fold higher PSC expansion compared to uncoated microcarriers (Chen A.K.L. et al., 2011). Recently, dissolvable microcarriers have been developed, which allow the retrieval of cells without using enzymatic dissociation (Badenes et al., 2014; Shekaran et al., 2016; Rodrigues et al., 2019).

By leveraging the self-aggregation property of PSCs, suspension-based cell culture systems are being developed to improve yield. Such systems promote cell-cell interactions while inhibiting the cell-matrix interactions. Usually, such systems consist of single cell culture in the presence of rho kinase (ROCK) inhibitor (Olmer et al., 2010; Abbasalizadeh et al., 2012), which supports long-term PSC survival in an undifferentiated state. PSCs grow in a monoclonal fashion and form suspended spheroids of varying sizes. Microfabrication technology has been used to further improve the homogeneity of PSC colonies (Hsiao and Palecek, 2012; Hookway et al., 2016). Optimization of bioreactor hydrodynamic conditions using combinations of static and stirred culture has enabled size-controlled aggregates of hPSCs (Abbasalizadeh et al., 2012). Traditionally, the yield of hPSCs is lower compared to mPSCs in suspension bioreactor cultures (Lipsitz et al., 2018). Recently, Lipsitz et al. found that the use of naïve hPSCs, as opposed to primed hPSCs, was a critical element for enabling high-yield expansion of PSCs (Over all 25-fold expansion; up to 5.7-fold higher compared to primed hPSCs) in suspension culture (Lipsitz et al., 2018). Despite tremendous progress in suspension-based cell culture, more research is needed in maintaining the homogeneity of cell aggregates in scalable suspension culture. Additionally, cells on the surface of the suspension aggregates experience uncontrolled shear stress, which could also lead to heterogeneous cell populations, as shear stress is known to affect stem cell fate (Toh and Voldman, 2011; Vining and Mooney, 2017).

## Conclusion and Future Outlook

Conventional methods of PSC expansion have clear and significant limitations in expansion. For development of large scale, defined and xeno-free PSC expansion systems, research should look toward using approaches with mechanobiological principles and 3D strategies for enhancing cell pluripotency retention and proliferation to improve current xeno-free expansion systems. Despite great progress in these fields, studying each physical cue in isolation is difficult as they are interconnected. It is challenging to draw conclusions regarding the effects of topography and stiffness due to many variations in study parameters, while 3D culture systems have much to be optimized. Nonetheless, these non-conventional methods have shown to improve PSC yield in xeno-free systems and thus should continue to be studied. Additionally, studies suggest that mechanobiological cues used with current PSC culture methods can enhance current PSC culture methods. The knowledge obtained in organoid cultures, mechanobiology, new advances in microfabrication and stimulus-responsive materials could contribute to future development of non-conventional systems for scaling up PSC expansion and revolutionize the field of regenerative medicine.

## Author Contributions

Literature search was conducted by SC and MR. Manuscript writing and editing were performed by SC, MR, and EY.

## Conflict of Interest

The authors declare that the research was conducted in the absence of any commercial or financial relationships that could be construed as a potential conflict of interest.

## References

[B1] Abagnale G. Sechi A. Steger M. Zhou Q. Kuo C. C. Aydin G. (2017). Surface topography guides morphology and spatial patterning of induced pluripotent stem cell colonies. *Stem Cell Rep.* 9 654–666. 10.1016/j.stemcr.2017.06.016 28757164 PMC5550028

[B2] Abbasalizadeh S. Larijani M. R. Samadian A. Baharvand H. (2012). Bioprocess development for mass production of size-controlled human pluripotent stem cell aggregates in stirred suspension bioreactor. *Tissue Eng. Part C Methods* 18 831–851. 10.1089/ten.tec.2012.0161 22559864

[B3] Akasaka T. Yokoyama A. Matsuoka M. Hashimoto T. Watari F. (2011). Maintenance of hemiround colonies and undifferentiated state of mouse induced pluripotent stem cells on carbon nanotube-coated dishes. *Carbon N. Y.* 49 2287–2299. 10.1016/j.carbon.2011.01.061

[B4] Alberti K. Davey R. E. Onishi K. George S. Salchert K. Seib F. P. (2008). Functional immobilization of signaling proteins enables control of stem cell fate. *Nat. Methods* 5 645–650. 10.1038/nmeth.1222 18552855

[B5] Amit M. Itskovitz-Eldor J. (2006). Feeder-free culture of human embryonic stem cells. *Methods Enzymol.* 420 37–49. 10.1016/S0076-6879(06)20003-X 17161692

[B6] Amit M. Margulets V. Segev H. Shariki K. Laevsky I. Coleman R. (2004). Human feeder layers for human embryonic stem cells. *Biol. Reprod.* 68 2150–2156. 10.1095/biolreprod.102.012583 12606388

[B7] Ankam S. Lim C. K. Yim E. K. F. (2015). Actomyosin contractility plays a role in MAP2 expression during nanotopography-directed neuronal differentiation of human embryonic stem cells. *Biomaterials* 47 20–28. 10.1016/j.biomaterials.2015.01.003 25682157

[B8] Ankam S. Suryana M. Chan L. Y. Moe A. A. K. Teo B. K. K. Law J. B. K. (2013). Substrate topography and size determine the fate of human embryonic stem cells to neuronal or glial lineage. *Acta Biomater.* 9 4535–4545. 10.1016/j.actbio.2012.08.018 22906625

[B9] Ankam S. Teo B. K. K. Pohan G. Ho S. W. L. Lim C. K. Yim E. K. F. (2018). Temporal changes in nucleus morphology, Lamin A/C and histone methylation during nanotopography-induced neuronal differentiation of stem cells. *Front. Bioeng. Biotechnol.* 6:69. 10.3389/fbioe.2018.00069 29904629 PMC5990852

[B10] Argentati C. Morena F. Tortorella I. Bazzucchi M. Porcellati S. Emiliani C. (2019). Insight into mechanobiology: how stem cells feel mechanical forces and orchestrate biological functions. *Int. J. Mol. Sci.* 20:5337. 10.3390/ijms20215337 31717803 PMC6862138

[B11] Badenes S. M. Fernandes T. G. Rodrigues C. A. V. Diogo M. M. Cabral Joaquim M. S. (2014). “Scalable Expansion of Human-Induced Pluripotent Stem Cells in Xeno-Free Microcarriers,” in *Stem Cells and Good Manufacturing Practices. Methods in Molecular Biology*, Vol. 1283 ed. Turksen K. (New York, NY: Humana Press). 10.1007/7651_2014_106 25108454

[B12] Bae D. Moon S. H. Park B. G. Park S. J. Jung T. Kim J. S. (2014). Nanotopographical control for maintaining undifferentiated human embryonic stem cell colonies in feeder free conditions. *Biomaterials* 35 916–928. 10.1016/j.biomaterials.2013.10.031 24183167

[B13] Baghbaderani B. A. Tian X. Cadet J. S. Shah K. Walde A. Tran H. (2016). A newly defined and xeno-free culture medium supports every-other-day medium replacement in the generation and long-term cultivation of human pluripotent stem cells. *PLoS One* 11:e0161229. 10.1371/journal.pone.0161229 27606941 PMC5016087

[B14] Banerjee I. Kumta P. Richardson T. (2018). *Peptide Conjugated Hydrogel Substrate for the Maintenance and Expansion of Human Pluripotent Stem Cells.* U.S. Patent No US 2018/0171286 A1 Washington, DC: United States Patent and Trademark Office.

[B15] Bangalore M. P. Adhikarla S. Mukherjee O. Panicker M. M. (2017). Genotoxic effects of culture media on human pluripotent stem cells. *Sci. Rep.* 7 1–12. 10.1038/srep42222 28176872 PMC5297241

[B16] Beattie G. M. Lopez A. D. Bucay N. Hinton A. Firpo M. T. King C. C. (2005). Activin a maintains pluripotency of human embryonic stem cells in the absence of feeder layers. *Stem Cells* 23 489–495. 15790770 10.1634/stemcells.2004-0279

[B17] Bedford P. Jy J. Collins L. Keizer S. (2018). Considering cell therapy product “Good Manufacturing Practice” status. *Front. Med.* 5:118. 10.3389/fmed.2018.00118 29761103 PMC5936751

[B18] Blin G. Lablack N. Louis-Tisserand M. Nicolas C. Picart C. Pucéat M. (2010). Nano-scale control of cellular environment to drive embryonic stem cells selfrenewal and fate. *Biomaterials* 31 1742–1750. 10.1016/j.biomaterials.2009.11.055 19962191

[B19] Brafman D. A. Chang C. W. Fernandez A. Willert K. Varghese S. Chien S. (2010). Long-term human pluripotent stem cell self-renewal on synthetic polymer surfaces. *Biomaterials* 31 9135–9144. 10.1016/j.biomaterials.2010.08.007 20817292 PMC2949524

[B20] Caliari S. R. Burdick J. A. (2016). A practical guide to hydrogels for cell culture. *Nat. Methods* 13 405–414. 10.1038/nmeth.3839 27123816 PMC5800304

[B21] Chan L. Y. Birch W. R. Yim E. K. F. Choo A. B. H. (2013). Temporal application of topography to increase the rate of neural differentiation from human pluripotent stem cells. *Biomaterials* 34 382–392. 10.1016/j.biomaterials.2012.09.033 23083932

[B22] Chen A. K. L. Chen X. Choo A. B. H. Reuveny S. Oh S. K. W. (2011). Critical microcarrier properties affecting the expansion of undifferentiated human embryonic stem cells. *Stem Cell Res.* 7 97–111. 10.1016/j.scr.2011.04.007 21763618

[B23] Chen G. Gulbranson D. R. Hou Z. Bolin J. M. Probasco M. D. Smuga-otto K. (2011). Chemically defined conditions for human iPS cell derivation and culture. *Nat. Methods* 8 424–429. 10.1038/nmeth.1593 21478862 PMC3084903

[B24] Chen G. Y. Pang D. W. P. Hwang S. M. Tuan H. Y. Hu Y. C. (2012). A graphene-based platform for induced pluripotent stem cells culture and differentiation. *Biomaterials* 33 418–427. 10.1016/j.biomaterials.2011.09.071 22014460

[B25] Chen K. G. Mallon B. S. McKay R. D. G. Robey P. G. (2014). Human pluripotent stem cell culture: considerations for maintenance, expansion, and therapeutics. *Cell Stem Cell* 14 13–26. 10.1016/j.stem.2013.12.005 24388173 PMC3915741

[B26] Chen W. Villa-Diaz L. G. Sun Y. Weng S. Kim J. K. Lam R. H. W. (2012). Nanotopography influences adhesion, spreading, and self-renewal of human embryonic stem cells. *ACS Nano* 6 4094–4103. 10.1021/nn3004923 22486594 PMC3358529

[B27] Chowdhury F. Li Y. Poh Y. C. Yokohama-Tamaki T. Wang N. Tanaka T. S. (2010a). Soft substrates promote homogeneous self-renewal of embryonic stem cells via downregulating cell-matrix tractions. *PLoS One* 5:e15655. 10.1371/journal.pone.0015655 21179449 PMC3001487

[B28] Chowdhury F. Na S. Li D. Poh Y. Tanaka T. S. Wang F. (2010b). Cell material property dictates stress-induced spreading and differentiation in embryonic stem cells. *Nat. Mater.* 9 82–88. 10.1038/nmat2563.Cell 19838182 PMC2833279

[B29] Conner D. A. (2000). Mouse embryo fibroblast (MEF) feeder cell preparation. *Curr. Protoc. Mol. Biol.* 51 23.2.1–23.2.7. 10.1002/0471142727.mb2302s51 18265203

[B30] Dakhore S. Nayer B. Hasegawa K. (2018). Human pluripotent stem cell culture: current status, challenges, and advancement. *Stem Cells Int.* 2018:7396905. 10.1155/2018/7396905 30595701 PMC6282144

[B31] Davidson K. C. Mason E. A. Pera M. F. (2015). The pluripotent state in mouse and human. *Development* 142 3090–3099. 10.1242/dev.116061 26395138

[B32] De Sousa P. A. Downie J. M. Tye B. J. Bruce K. Dand P. Dhanjal S. (2016). Development and production of good manufacturing practice grade human embryonic stem cell lines as source material for clinical application. *Stem Cell Res.* 17 379–390. 10.1016/j.scr.2016.08.011 27639108

[B33] Dvorak P. Dvorakova D. Hampl A. (2006). Fibroblast growth factor signaling in embryonic and cancer stem cells. *FEBS Lett.* 580 2869–2874. 10.1016/j.febslet.2006.01.095 16516203

[B34] Evans N. Minelli C. Gentleman E. LaPointe V. Patankar S. Kallivretaki M. (2009). Substrate stiffness affects early differentiation events in embryonic stem cells. *Eur. Cells Mater.* 18 1–14. 10.22203/ecm.v018a01 19768669

[B35] Eyckmans J. Boudou T. Yu X. Chen C. S. (2011). A Hitchhiker’s guide to mechanobiology. *Dev. Cell* 21 35–47. 21763607 10.1016/j.devcel.2011.06.015 PMC3155761

[B36] Fan Y. Wu J. Ashok P. Hsiung M. Tzanakakis E. S. (2015). Production of human pluripotent stem cell therapeutics under defined xeno-free conditions: progress and challenges. *Stem Cell Rev. Rep.* 11 96–109. 10.1007/s12015-014-9544-x 25077810 PMC4312540

[B37] Hammad M. Rao W. Smith J. G. W. Anderson D. G. Langer R. Young L. E. (2016). Identification of polymer surface adsorbed proteins implicated in pluripotent human embryonic stem cell expansion. *Biomater. Sci.* 4 1381–1391. 10.1039/c6bm00214e 27466628 PMC5038343

[B38] Hashemi S. M. Soudi S. Shabani I. Naderi M. Soleimani M. (2011). The promotion of stemness and pluripotency following feeder-free culture of embryonic stem cells on collagen-grafted 3-dimensional nanofibrous scaffold. *Biomaterials* 32 7363–7374. 10.1016/j.biomaterials.2011.06.048 21762983

[B39] Hayashi Y. Furue M. K. (2016). Biological effects of culture substrates on human pluripotent stem cells. *Stem Cells Int.* 2016:5380560. 10.1155/2016/5380560 27656216 PMC5021488

[B40] Hey C. A. B. Saltõkova K. B. Bisgaard H. C. Møller L. B. (2018). Comparison of two different culture conditions for derivation of early hiPSC. *Cell Biol. Int.* 42 1467–1473. 10.1002/cbin.10966 29603519

[B41] Hookway T. A. Butts J. C. Lee E. Tang H. McDevitt T. C. (2016). Aggregate formation and suspension culture of human pluripotent stem cells and differentiated progeny. *Methods* 101 11–20. 10.1016/j.ymeth.2015.11.027 26658353

[B42] Hovatta O. Mikkola M. Gertow K. Strömberg A. M. Inzunza J. Hreinsson J. (2003). A culture system using human foreskin fibroblasts as feeder cells allows production of human embryonic stem cells. *Hum. Reprod.* 18 1404–1409. 10.1093/humrep/deg290 12832363

[B43] Hsiao C. Palecek S. P. (2012). Microwell regulation of pluripotent stem cell self-renewal and differentiation. *Bionanoscience* 2 266–276. 23483802 10.1007/s12668-012-0050-9 PMC3589576

[B44] Huang Y. Osorno R. Tsakiridis A. Wilson V. (2012). In Vivo differentiation potential of epiblast stem cells revealed by chimeric embryo formation. *Cell Rep.* 2 1571–1578. 10.1016/j.celrep.2012.10.022 23200857

[B45] Ireland R. G. Simmons C. A. (2015). Human pluripotent stem cell mechanobiology: Manipulating the biophysical microenvironment for regenerative medicine and tissue engineering applications. *Stem Cells* 33 3187–3196. 10.1002/stem.2105 26189759

[B46] Jaggy M. Zhang P. Greiner A. M. Autenrieth T. J. Nedashkivska V. Efremov A. N. (2015). Hierarchical micro-nano surface topography promotes long-term maintenance of undifferentiated mouse embryonic stem cells. *Nano Lett.* 15 7146–7154. 10.1021/acs.nanolett.5b03359 26351257

[B47] James D. Levine A. J. Besser D. Hemmati-Brivanlou A. (2005). TGFβ/activin/nodal signaling is necessary for the maintenance of pluripotency in human embryonic stem cells. *Development* 132 1273–1282. 10.1242/dev.01706 15703277

[B48] Jeon K. Oh H. J. Lim H. Kim J. H. Lee D. H. Lee E. R. (2012). Self-renewal of embryonic stem cells through culture on nanopattern polydimethylsiloxane substrate. *Biomaterials* 33 5206–5220. 10.1016/j.biomaterials.2012.04.011 22541355

[B49] Ji L. Lapointe V. L. S. Evans N. D. Stevens M. M. (2012). Changes in embryonic stem cell colony morphology and early differentiation markers driven by colloidal crystal topographical cues. *Eur. Cells Mater.* 23 135–146. 10.22203/eCM.v023a10 22370796

[B50] Johnson B. V. Shindo N. Rathjen P. D. Rathjen J. Keough R. A. (2008). Understanding pluripotency – How embryonic stem cells keep their options open. *Mol. Hum. Reprod.* 14 513–520. 10.1093/molehr/gan048 18716052

[B51] Kaufman M. H. Evans M. J. (1981). Establishment in culture of pluripotential cells from mouse embryos. *Nature* 292 154–156. 7242681 10.1038/292154a0

[B52] Keung A. J. Asuri P. Kumar S. Schaffer D. V. (2012). Soft microenvironments promote the early neurogenic differentiation but not self-renewal of human pluripotent stem cells. *Integr. Biol.* 4 1049–1058. 10.1039/c2ib20083j 22854634 PMC3459311

[B53] Khetan S. Guvendiren M. Legant W. R. Cohen D. M. Chen C. S. Burdick J. A. (2013). Degradation-mediated cellular traction directs stem cell fate in covalently crosslinked three-dimensional hydrogels. *Nat. Mater.* 12 458–465. 10.1038/nmat3586 23524375 PMC3633615

[B54] Kim I. G. Gil C. H. Seo J. Park S. J. Subbiah R. Jung T. H. (2018). Mechanotransduction of human pluripotent stem cells cultivated on tunable cell-derived extracellular matrix. *Biomaterials* 150 100–111. 10.1016/j.biomaterials.2017.10.016 29035736

[B55] Kimura Y. Kasai K. Miyata S. (2018). Feeder-free culture for mouse induced pluripotent stem cells by using UV/ozone surface-modified substrates. *Mater. Sci. Eng. C* 92 280–286. 10.1016/j.msec.2018.06.053 30184752

[B56] Ko J.-Y. Oh H.-J. Lee J. Im G.-I. (2017). Nanotopographic influence on the in vitro behavior of induced pluripotent stem cells. *Tissue Eng. Part A* 24 595–606. 10.1089/ten.tea.2017.0144 28726546

[B57] Kong Y. P. Tu C. H. Donovan P. J. Yee A. F. (2013). Expression of Oct4 in human embryonic stem cells is dependent on nanotopographical configuration. *Acta Biomater.* 9 6369–6380. 10.1016/j.actbio.2013.01.036 23391989

[B58] Le M. N. T. Hasegawa K. (2019). Expansion culture of human pluripotent stem cells and production of cardiomyocytes. *Bioengineering* 6:48. 10.3390/bioengineering6020048 31137703 PMC6632060

[B59] Lee S. Stanton A. E. Tong X. Yang F. (2019). Hydrogels with enhanced protein conjugation efficiency reveal stiffness-induced YAP localization in stem cells depends on biochemical cues. *Biomaterials* 202 26–34. 10.1016/j.biomaterials.2019.02.021 30826537 PMC6447317

[B60] Lei Y. Schaffer D. V. (2013). A fully defined and scalable 3D culture system for human pluripotent stem cell expansion and differentiation. *Proc. Natl. Acad. Sci. U.S.A.* 10 E5039–E5048. 10.1073/pnas.1309408110 24248365 PMC3876251

[B61] Li L. Bennett S. A. L. Wang L. (2012). Role of E-cadherin and other cell adhesion molecules in survival and differentiation of human pluripotent stem cells. *Cell Adhes. Migr.* 6 59–70. 10.4161/cam.6.1.19583 22647941 PMC3364139

[B62] Lipsitz Y. Y. Woodford C. Yin T. Hanna J. H. Zandstra P. W. (2018). Modulating cell state to enhance suspension expansion of human pluripotent stem cells. *Proc. Natl. Acad. Sci. U.S.A.* 115 6369–6374. 10.1073/pnas.1714099115 29866848 PMC6016797

[B63] Liu L. Yoshioka M. Nakajima M. Ogasawara A. Liu J. Hasegawa K. (2014). Nanofibrous gelatin substrates for long-term expansion of human pluripotent stem cells. *Biomaterials* 35 6259–6267. 10.1016/j.biomaterials.2014.04.024 24811263

[B64] Llames S. García-Pérez E. Meana Á Larcher F. del Río M. (2015). Feeder layer cell actions and applications. *Tissue Eng. Part B Rev.* 21 345–353. 10.1089/ten.teb.2014.0547 25659081 PMC4533020

[B65] López-Fagundo C. Livi L. L. Ramchal T. Darling E. M. Hoffman-Kim D. (2016). A biomimetic synthetic feeder layer supports the proliferation and self-renewal of mouse embryonic stem cells. *Acta Biomater.* 39 55–64. 10.1016/j.actbio.2016.04.047 27142253 PMC4905775

[B66] Lü D. Luo C. Zhang C. Li Z. Long M. (2014). Differential regulation of morphology and stemness of mouse embryonic stem cells by substrate stiffness and topography. *Biomaterials* 35 3945–3955. 10.1016/j.biomaterials.2014.01.066 24529627

[B67] Lyu Z. Wang H. Wang Y. Ding K. Liu H. Yuan L. (2014). Maintaining the pluripotency of mouse embryonic stem cells on gold nanoparticle layers with nanoscale but not microscale surface roughness. *Nanoscale* 6 6959–6969. 10.1039/c4nr01540a 24839204

[B68] Macgregor M. Williams R. Downes J. Bachhuka A. Vasilev K. (2017). The role of controlled surface topography and chemistry on mouse embryonic stem cell attachment, growth and self-renewal. *Materials (Basel)* 10:1081. 10.3390/ma10091081 28906470 PMC5615735

[B69] Madl C. M. Lesavage B. L. Dewi R. E. Dinh C. B. Stowers R. S. Khariton M. (2017). Maintenance of neural progenitor cell stemness in 3D hydrogels requires matrix remodelling. *Nat. Mater.* 16 1233–1242. 10.1038/nmat5020 29115291 PMC5708569

[B70] Maldonado M. Wong L. Y. Echeverria C. Ico G. Low K. Fujimoto T. (2015). The effects of electrospun substrate-mediated cell colony morphology on the self-renewal of human induced pluripotent stem cells. *Biomaterials* 50 10–19. 10.1016/j.biomaterials.2015.01.037 25736491

[B71] Markert L. D. Lovmand J. Foss M. Lauridsen R. H. Lovmand M. Füchtbauer E.-M. (2009). Identification of distinct topographical surface microstructures favoring either undifferentiated expansion or differentiation of murine embryonic stem cells. *Stem Cells Dev.* 18 1331–1342. 10.1089/scd.2009.0114 19508153

[B72] Meade K. A. White K. J. Pickford C. E. Holley R. J. Marson A. Tillotson D. (2013). Immobilization of heparan sulfate on electrospun meshes to support embryonic stem cell culture and differentiation. *J. Biol. Chem.* 288 5530–5538. 10.1074/jbc.M112.423012 23235146 PMC3581394

[B73] Mih J. D. Sharif A. S. Liu F. Marinkovic A. Symer M. M. Tschumperlin D. J. (2011). A multiwell platform for studying stiffness-dependent cell biology. *PLoS One* 6:e19929. 10.1371/journal.pone.0019929 21637769 PMC3103526

[B74] Mosiewicz K. A. Kolb L. Van Der Vlies A. J. Martino M. M. Lienemann P. S. Hubbell J. A. (2013). In situ cell manipulation through enzymatic hydrogel photopatterning. *Nat. Mater.* 12 1072–1078. 10.1038/nmat3766 24121990

[B75] Musah S. Morin S. A. Wrighton P. J. Zwick D. B. Jin S. Kiessling L. L. (2012). Glycosaminoglycan-binding hydrogels enable mechanical control of human pluripotent stem cell self-renewal. *ACS Nano* 6 10168–10177. 10.1021/nn3039148 23005914 PMC3509190

[B76] Nichols J. Smith A. (2009). Naive and primed pluripotent states. *Cell Stem Cell* 4 487–492. 10.1016/j.stem.2009.05.015 19497275

[B77] Nur-E-Kamal A. Ahmed I. Kamal J. Schindler M. Meiners S. (2005). Three-dimensional nanofibrillar surfaces promote self-renewal in mouse embryonic stem cells. *Stem Cells* 24 426–433. 16150921 10.1634/stemcells.2005-0170

[B78] Olmer R. Haase A. Merkert S. Cui W. Paleèek J. Ran C. (2010). Long term expansion of undifferentiated human iPS and ES cells in suspension culture using a defined medium. *Stem Cell Res.* 5 51–64. 10.1016/j.scr.2010.03.005 20478754

[B79] Pelham R. J. Wang Y. L. (1997). Cell locomotion and focal adhesions are regulated by substrate flexibility. *Proc. Natl. Acad. Sci. U.S.A.* 94 13661–13665. 10.1073/pnas.94.25.13661 9391082 PMC28362

[B80] Price A. J. Huang E. Y. Sebastiano V. Dunn A. R. (2017). A semi-interpenetrating network of polyacrylamide and recombinant basement membrane allows pluripotent cell culture in a soft, ligand-rich microenvironment. *Biomaterials* 121 179–192. 10.1016/j.biomaterials.2016.12.005 28088685

[B81] Pryzhkova M. V. Aria I. Cheng Q. Harris G. M. Zan X. Gharib M. (2014). Carbon nanotube-based substrates for modulation of human pluripotent stem cell fate. *Biomaterials* 35 5098–5109. 10.1016/j.biomaterials.2014.03.011 24690530 PMC4943838

[B82] Przybyla L. Lakins J. N. Weaver V. M. (2016). Tissue mechanics orchestrate wnt-dependent human embryonic stem cell differentiation. *Cell Stem Cell* 19 462–475. 10.1016/j.stem.2016.06.018 27452175 PMC5336327

[B83] Reimer A. Vasilevich A. Hulshof F. Viswanathan P. Van Blitterswijk C. A. De Boer J. (2016). Scalable topographies to support proliferation and Oct4 expression by human induced pluripotent stem cells. *Sci. Rep.* 6:18948. 10.1038/srep18948 26757610 PMC4725348

[B84] Richards M. Fong C. Y. Chan W. K. Wong P. C. Bongso A. (2002). Human feeders support prolonged undifferentiated growth of human inner cell masses and embryonic stem cells. *Nat. Biotechnol.* 20 933–936. 10.1038/nbt726 12161760

[B85] Rodrigues A. L. Rodrigues C. A. V. Gomes A. R. Vieira S. F. Badenes S. M. Diogo M. M. (2019). Dissolvable microcarriers allow scalable expansion and harvesting of human induced pluripotent stem cells under xeno-free conditions. *Biotechnol. J.* 14 1–12. 10.1002/biot.201800461 30320457

[B86] Rys J. P. Monteiro D. A. Alliston T. (2016). Mechanobiology of TGFβ signaling in the skeleton. *Matrix Biol.* 5 413–425. 10.1016/j.matbio.2016.02.002 26877077 PMC4875828

[B87] Saha K. Mei Y. Reisterer C. M. Pyzocha N. K. Yang J. Muffat J. (2011). Surface-engineered substrates for improved human pluripotent stem cell culture under fully defined conditions. *Proc. Natl. Acad. Sci. U.S.A.* 108 18714–18719. 10.1073/pnas.1114854108 22065768 PMC3219112

[B88] Shekaran A. Lam A. Sim E. Jialing L. Jian L. Wen J. T. P. (2016). Biodegradable ECM-coated PCL microcarriers support scalable human early MSC expansion and in vivo bone formation. *Cytotherapy* 18 1332–1344. 10.1016/j.jcyt.2016.06.016 27503763

[B89] Smith A. G. Heath J. K. Donaldson D. D. Wong G. G. Moreau J. Stahl M. (1988). Inhibition of pluripotential embryonic stem cell differentiation by purified polypeptides. *Nature* 336 688–690. 3143917 10.1038/336688a0

[B90] Sohi A. N. Naderi-Manesh H. Soleimani M. Yasaghi E. R. Manjili H. K. Tavaddod S. (2018). Synergistic effect of co-immobilized FGF-2 and vitronectin-derived peptide on feeder-free expansion of induced pluripotent stem cells. *Mater. Sci. Eng. C* 93 157–169. 10.1016/j.msec.2018.07.072 30274048

[B91] Srinivasan A. Toh Y. C. Loh X. J. Toh W. S. (2016). Substrates and surfaces for control of pluripotent stem cell fate and function. *Adv. Surfaces Stem Cell Res.* 343–380. 10.1002/9781119242642.ch12 30430755

[B92] Sun Y. Villa-Diaz L. G. Lam R. H. W. Chen W. Krebsbach P. H. Fu J. (2012). Mechanics regulates fate decisions of human embryonic stem cells. *PLoS One* 7:e37178. 10.1371/journal.pone.0037178 22615930 PMC3353896

[B93] Sung T.-C. Li H.-F. Higuchi A. Ling Q.-D. Yang J.-S. Tseng Y.-C. Pan C. H. P. (2018). Human pluripotent stem cell culture on polyvinyl alcohol-co-itaconic acid hydrogels with varying stiffness under xeno-free conditions. *J. Vis. Exp.* 132:e57314. 10.3791/57314 29443075 PMC5912358

[B94] Takahashi K. Narita M. Yokura M. Ichisaka T. Yamanaka S. (2009). Human induced pluripotent stem cells on autologous feeders. *PLoS One* 4:e8067. 10.1371/journal.pone.0008067 19956543 PMC2780725

[B95] Takahashi K. Tanabe K. Ohnuki M. Narita M. Ichisaka T. Tomoda K. (2007). Induction of pluripotent stem cells from adult human fibroblasts by defined factors. *Cell* 131 861–872. 10.1016/j.cell.2007.11.019 18035408

[B96] Takahashi K. Yamanaka S. (2006). Induction of pluripotent stem cells from mouse embryonic and adult fibroblast cultures by defined factors. *Cell* 126 663–676. 10.1016/j.cell.2006.07.024 16904174

[B97] Thomson J. A. Itskovitz-Eldor J. Shapiro S. S. Waknitz M. A. J. Swiergiel J. J. Marshall V. S. (1998). Embryonic stem cell lines derived from human blastocysts. *Science* 282 1145–1147. 10.1126/science.282.5391.1145 9804556

[B98] Toh Y. C. Voldman J. (2011). Fluid shear stress primes mouse embryonic stem cells for differentiation in a self-renewing environment via heparan sulfate proteoglycans transduction. *FASEB J.* 25 1208–1217. 10.1096/fj.10-168971 21183594 PMC3058703

[B99] Tomishima M. (2014). Conditioning pluripotent stem cell media with mouse embryonic fibroblasts (MEF-CM). *StemBook* 20:2. 10.3824/stembook.1.68.1 23658989

[B100] Villa-Diaz L. G. Ross A. M. Lahann J. Krebsbach P. H. (2013). The evolution of human pluripotent stem cell culture: from feeder cells to synthetic coatings. *Stem Cells* 31 1–7. 10.1002/stem.1260 23081828 PMC3537180

[B101] Vining K. H. Mooney D. J. (2017). Mechanical forces direct stem cell behaviour in development and regeneration. *Nat. Rev. Mol. Cell Biol.* 18 728–742. 10.1016/j.bone.2016.06.013 29115301 PMC5803560

[B102] Wang P. Y. Khan S. Nguyen T. Kingshott P. Wong R. C. B. (2018). Topographical modulation of pluripotency and differentiation of human embryonic stem cells. *IEEE Trans. Nanotechnol.* 17 381–384. 10.1109/TNANO.2017.2763604

[B103] Williams R. L. Hilton D. J. Pease S. Willson T. A. Stewart C. L. Gearing D. P. (1988). Myeloid leukaemia inhibitory factor maintains the developmental potential of embryonic stem cells. *Nature* 336 684–687. 10.1038/336684a0 3143916

[B104] Yang H. Qiu Y. Zeng X. Ding Y. Zeng J. Lu K. (2016). Effect of a feeder layer composed of mouse embryonic and human foreskin fibroblasts on the proliferation of human embryonic stem cells. *Exp. Ther. Med.* 11 2321–2328. 10.3892/etm.2016.3204 27313670 PMC4888025

[B105] Yang Y. Liu B. Xu J. Wang J. Wu J. Shi C. (2017). Derivation of pluripotent stem cells with in vivo embryonic and extraembryonic potency. *Cell* 169 243.e25–257.e25. 10.1016/j.cell.2017.02.005 28388409 PMC5679268

[B106] Yasuda S. Y. Ikeda T. Shahsavarani H. Yoshida N. Nayer B. Hino M. (2018). Chemically defined and growth-factor-free culture system for the expansion and derivation of human pluripotent stem cells. *Nat. Biomed. Eng.* 2 173–182. 10.1038/s41551-018-0200-7 31015717

[B107] Ying Q. L. Wray J. Nichols J. Batlle-Morera L. Doble B. Woodgett J. (2008). The ground state of embryonic stem cell self-renewal. *Nature* 453 519–523. 10.1038/nature06968 18497825 PMC5328678

[B108] Yu J. Smuga-Otto K. Antosiewicz-Bourget J. Frane J. L. Thomson J. A. Vodyanik M. A. (2007). Induced pluripotent stem cell lines derived from human somatic cells. *Science* 318 1917–1920. 10.1126/science.1151526 18029452

[B109] Zandén C. Hellström Erkenstam N. Padel T. Wittgenstein J. Liu J. Kuhn H. G. (2014). Stem cell responses to plasma surface modified electrospun polyurethane scaffolds. *Nanomedicine* 10 e949–e958. 10.1016/j.nano.2014.01.010 24524929

[B110] Zimmerlin L. Park T. S. Huo J. S. Verma K. Pather S. R. Talbot C. C. (2016). Tankyrase inhibition promotes a stable human naïve pluripotent state with improved functionality. *Development* 143 4368–4380. 10.1242/dev.138982 27660325 PMC5201042

